# Child saliva microbiota and caries: a randomized controlled maternal education trial in rural Uganda

**DOI:** 10.1038/s41598-022-11979-y

**Published:** 2022-05-12

**Authors:** Grace K. M. Muhoozi, Kelvin Li, Prudence Atukunda, Anne B. Skaare, Tiril Willumsen, Morten Enersen, Ane C. Westerberg, Alison Morris, Alexandre R. Vieira, Per O. Iversen, Barbara A. Methé

**Affiliations:** 1 Department of Family Life and Consumer Studies, Kyambogo University, Kampala, Uganda; 2 Center for Medicine and the Microbiota, University of Pittsburgh, Pittsburgh, USA; 3 Department of Nutrition, Institute of Basic Medical Sciences, University of Oslo, Blindern, P.O. Box 1046, 0317 Oslo, Norway; 4 Institute of Clinical Dentistry, Institute of Oral Biology, Faculty, of Dentistry, University of Oslo, Oslo, Norway; 5 Institute of Oral Biology, Faculty, of Dentistry, University of Oslo, Oslo, Norway; 6 Institute of Health Sciences, Kristiania University College, Oslo, Norway; 7 Division of Obstetrics and Gynecology, Oslo University Hospital, Oslo, Norway; 8 Department of Medicine, Division of Pulmonary, Allergy and Critical Care Medicine, University of Pittsburgh, Pittsburgh, USA; 9 Department of Oral and Craniofacial Sciences Biology, School of Dental Medicine, University of Pittsburgh, Pittsburgh, USA; 10 Department of Haematology, Oslo University Hospital, Oslo, Norway; 11 Division of Human Nutrition, Stellenbosch University, Tygerberg, South Africa

**Keywords:** Microbiology, Health care, Medical research, Risk factors

## Abstract

Undernutrition is a public health challenge in sub-Saharan countries, including Uganda. In a previous randomized controlled trial (RCT) with a nutrition, hygiene and stimulation education intervention among mothers of 6 months’ old children, we found less caries in the intervention group when the children were 36 months of age. We now examined the effects of (i) the intervention on the microbiota, (ii) microbiota on caries, and (iii) the intervention and microbiota on caries. The original RCT comprised 511 mother/child pairs whereas in the current study we had access to data from 344/511 (67%) children aged 36 months. The saliva microbiota was determined using 16S rRNA gene sequencing. Carious lesions (a proxy for dental health) were identified using close-up intra-oral photographs of the upper front teeth. Statistical models were used to determine host-microbiota associations. The intervention had a significant effect on the microbiota, e.g. an increase in *Streptococcus* abundance and decreases in *Alloprevotella* and *Tannerella*. Significant associations between the microbiota and dental caries were identified: Positive associations of *Capnocytophaga* and *Tannerella* suggest that these taxa may be deleterious to dental health while negative associations of *Granulicatella*, *Fusobacterium*, and *Abiotrophia* suggest taxa potentially beneficial or benign contributors to dental health. Based on taxonomic profiles, the effects of the intervention and microbiota on dental health may be independent of one another. Educational interventions with emphasis on nutrition and oral hygiene may provide a feasible strategy to decrease progression of childhood caries in low-resource settings.

## Introduction

The United Nation Sustainable Development Goal 2 aims to end all forms of hunger by 2030. Although some improvements have been made, several low- and middle-income countries in sub-Saharan Africa show slow progress to reach this goal^1^. Moreover, there is increasing awareness that poor dental health is a major contributing factor to undernutrition, in particular in low- and middle-income countries^2,3^.

Dental caries, a multifactorial disease consisting of host, oral microbiota, and environmental impact, is one of the most frequent chronic infectious diseases of young children in low- and middle-income countries such as Uganda^4,5^. Caries forms through a complex interaction over time between biofilm formation and dietary consumption of simple sugars and fermentable carbohydrates that can lead to proliferation of acidogenic and aciduric bacteria in susceptible hosts^6,7^ Notably, these nutrients are frequent dietary components among impoverished children in several low- and middle-income countries (LMICs).

Educational interventions that focus on oral health may provide a feasible strategy to decrease development and progression of childhood caries in low- and middle-income countries. We therefore examined dental status among Ugandan children aged 36 months whose mothers participated in a randomized controlled trial (RCT) educating them in nutrition, sanitation and oral hygiene^8^. We found that the frequency of cleaning of the children’s teeth was approximately twice as high in the intervention as in the control group and cavitated carious lesions occurred more frequently in the control than the intervention group. However, there was no evidence of association between the occurrence of caries and impaired child linear growth (stunting, a marker of chronic undernutrition)^9^.

To clarify the possible role of the microbiota on the caries-reducing effects of this education intervention, we here examined the relation between saliva microbiota and caries. To this end we employed culture-independent 16S rRNA gene sequencing to determine microbiota profiles, which were subsequently used in multiple statistical models designed to identify relationships between microbiota, the educational intervention, and dental caries identified with photographs.

## Methods

### Study area and participants

The primary outcome of the original cluster-RCT was to reduce linear growth faltering. It was performed in Kabale and Kisoro districts in South-Western Uganda because of the high levels of child stunting there^10^. The original cluster-RCT included 511 children aged 6–8 months and is detailed elsewhere^8,11^ and in the Supplementary Methods. For the current study assessing dental health and saliva microbiota at child age 36 months, we included a randomized sub-sample of 344/511 (67%) of the children (outlined in the Supplementary Methods).

### Approvals

The study was approved by (i) Makerere University School of Public Health, Higher Degrees Research and Ethics Committee (IRB00011353), (ii) Uganda National Council for Science and Technology (No. TASOREC/06/15-UG-REC-009), (iii) Norwegian Regional Committee for Medical and Health Research Ethics (no. 2013/1833), and (iv) University of Pittsburgh Institutional Review Board (IRB # PRO16100564). The consent form document was translated into the local language for the parents/care-givers, and all of them gave written or thumb-printed, informed consent to participate. The trial was first registered with ClinicalTrials.gov ID NCT02098031 on 21/03/2014. All methods were performed in accordance with relevant guidelines and regulations.

### Delivery and content of the education intervention

The main education intervention started when the children were 6–8 months and lasted 6 months, and emphasized nutrition, oral hygiene and stimulation and was delivered to mothers in the intervention group as described in the Supplementary Methods.

### Oral hygiene promotion

The promotion of oral hygiene was as previously detailed^9^: When the children were 12–16 months and all had erupted at least four teeth (two upper and two lower incisors), the mothers in the intervention group were educated on the importance of good oral hygiene to prevent caries in their children. The children were given age-appropriate toothbrushes (but not toothpaste), and the nutrition educators demonstrated tooth-cleaning. The rest of the household also received toothbrushes to avoid sharing the index child’s toothbrush. Moreover, the mothers were given instructions to (i) brush the child’s teeth with clean, boiled and cooled water at least twice a day, especially before going to bed; (ii) clean the brushes after use before storing them safely in a clean container, preferably with a cover; and (iii) not to share the toothbrushes. Mothers were counselled to stop the habits of licking the children’s feeding utensils and chewing food/herbal medicine to spit in the baby’s mouth. During the follow-up period, the field-workers visited the mothers on three occasions to encourage them to continue these oral hygiene practices. Lost or damaged toothbrushes were replaced also during these visits.

### Collection of oral data

We took close-up intra-oral photographs of the upper front teeth of the children to determine the occurrence of carious lesions, registered as unmistakable cavities progressing into the dentine as recommended by World Health Organization^12–14^. The photographs were taken with a Canon EOS 1100D Camera (Canon Inc., Taiwan) using a 60 mm macro-lens and a macro-ring flash. We aimed at an aperture of F stop 22 for the sharpness of the picture. Carious lesions are defined as the occurrence of any signs of dental caries on any tooth surface^15^. However, as the early stages of dental caries are not possible to identify on photographs, only obvious, cavitated lesions into the dentine were registered as caries. The photographs of the upper front teeth (four incisors) were evaluated by two experienced dentists (A.B.S. and T.W.) who were blinded to the children’s group allocation. Inter-examiner agreement measured by kappa was 0.97. In case of disagreement, the tooth was scored as sound. Examples of such photographs are given in our previous report^9^. The fluoride content in various sources of drinking water was assayed as described^9^.

### Oral microbiota collection, sample processing and analysis

Saliva samples were collected using Omnigene oral kits (DNA Genotek, Ontario, Canada). DNA extraction was performed using the Qiagen DNeasy Powersoil Kit (Germantown, MD) and processed per manufacturer’s protocol. Reagent blanks were included as negative controls and cells from a microbial community of known composition (ZymoBiomics Microbial Community Standards; Zymo Research, Irvine, CA) as a positive control. The V4 region of the 16S rRNA gene was amplified and prepared for sequencing on an Illumina MiSeq platform as described in the Supplementary Methods.

### Microbiota and intervention models

**Figure 1:**
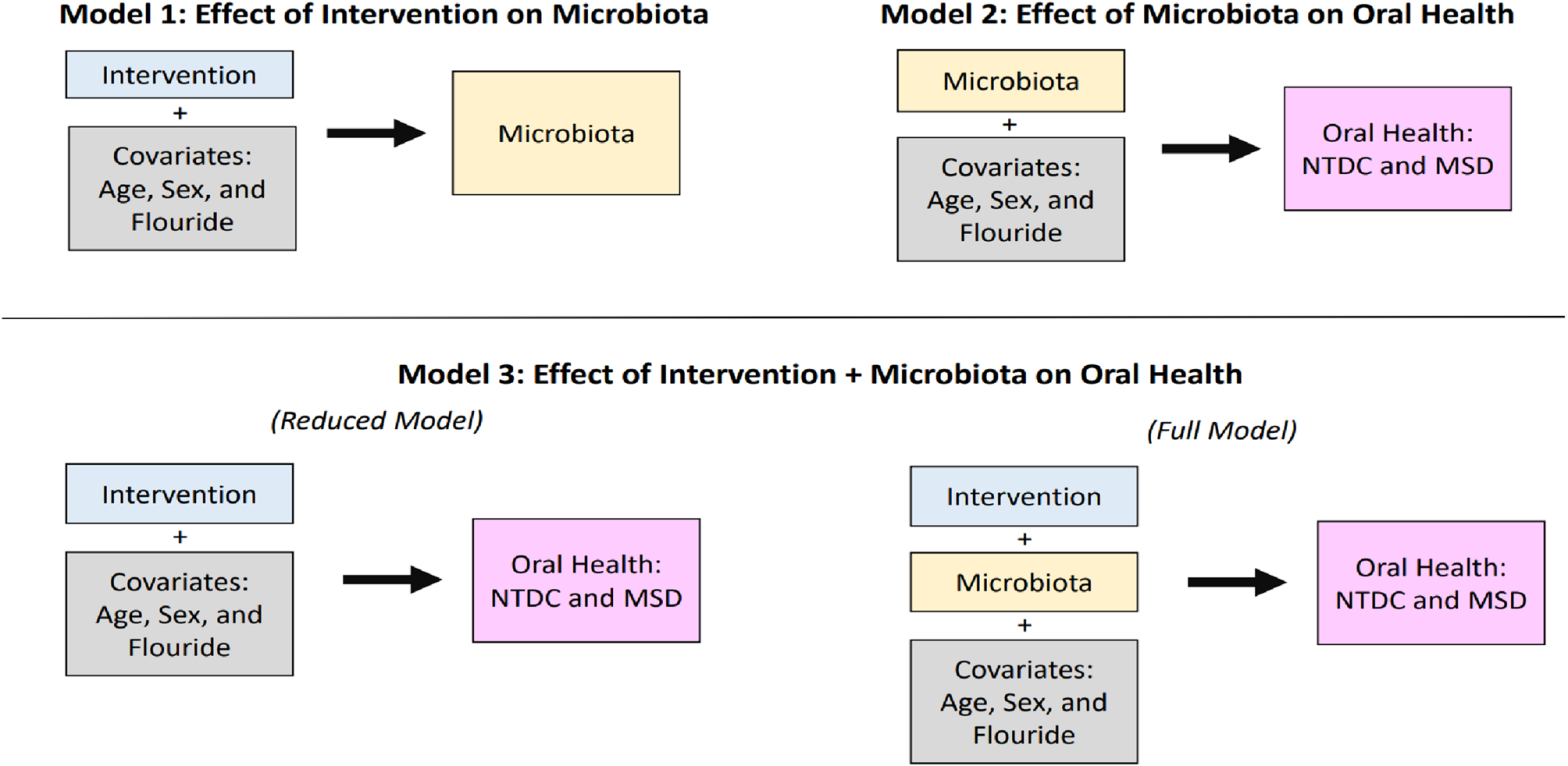
Statistical models used to analyze the interactions between intervention, the microbiota and oral (i.e. dental) health. The rectangles on the left- or right-hand side of the arrows represent groups of variables designated as either predictors (x) or response variables (y), respectively, in a linear regression model. Model 3 is evaluated by fitting the reduced and full model separately, then comparing them to determine whether inclusion of the microbiota improved the prediction of oral health. NTDC, number of dentin caries. MSD, most severe diagnosis.

We developed three linear models to examine the relationship between educational intervention, microbiota, and oral (dental) health (Fig. 1 and the Supplementary Methods). The variables included in models are the covariates of fluoride concentration in drinking water, sex, and age. Dental health status of the children was quantified by the “number of teeth with dentin caries” (NTDC) and “most severe diagnosis” (MSD) at 36 months identified with photographs. From the 16S rRNA gene sequence clustering and annotation pipeline 155 and 162 samples from the control and intervention groups, respectively, were generated and subsequently analyzed. In the effect of intervention on the microbiota model (i), microbiota metrics are considered responses to the educational intervention as a predictor (treatment). In the effect of microbiota on dental health model (ii), microbiota profiles are utilized along with the covariates (age, sex and fluoride) as predictors of caries at 36 months. In the effect of intervention and microbiota on caries model (iii), both the intervention variable, microbiota profiles and covariates are used so that the contributions of both factors can be cumulatively considered and to determine whether the inclusion of the microbiota profiles improves the predictability of dental health in contrast to intervention alone. We refer to the Supplementary Methods for descriptions of other statistical analyses and considerations for multiple testing.

## Results

### Recruitment of the mother/child pairs and their characteristics

**Figure 2:**
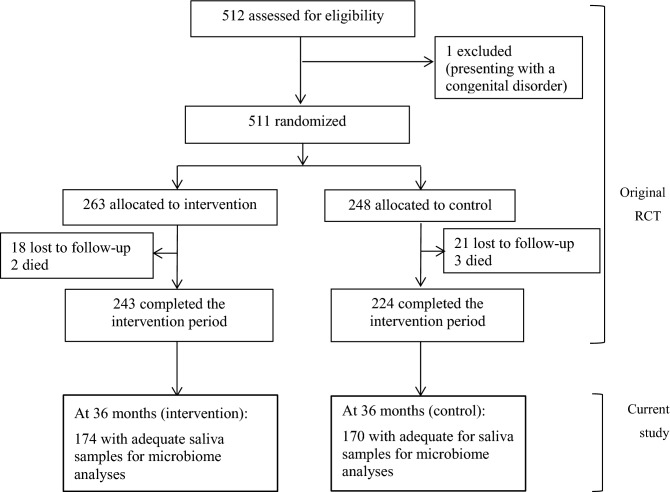
Flowchart showing the inclusion of study participants into the original trial and the current follow-up study. RCT, randomized controlled trial.

Figure 2 shows the step-by-step numbers of recruited participants according to time since enrollment into the original RCT. At 36 months of age, 72% and 76% of the children who completed the original trial had adequate saliva samples for microbiota analyses in the intervention and control group, respectively. Table 1 shows socio-demographic characteristics of the mother–child pairs obtained when the children were aged 6–8 months, in both the original cluster-RCT cohort and in the current study cohort. Notably, there were no significant differences in any characteristic between the two study groups (intervention and control) in either the original cluster-RCT or the current study cohort, except that more mothers in the current control group breastfed 8 or more times/day compared with the current intervention group. The prevalence of markers of poor nutritional status (i.e. stunting, underweight and wasting) were not significantly altered by the intervention, neither in the original cluster-RCT nor in the current study (Table 1). Moreover, no significant differences were noted among the intervention groups in the original cluster-RCT cohort versus that in the current study cohort, or among the control group in the original cluster-RCT versus that in the current study cohort. These results suggest that the current study cohort most likely was representative of the original cluster-RCT cohort.

**Table 1:** Study population characteristics at baseline (children aged 6–8 months).

Characteristic^1^	Original RCT		Current study	
	Intervention (*n* = 263)	Control (*n* = 248)	Intervention (*n* = 161–174)^3^	Control (*n* = 156–170)^3^
**Children**				
Males	139 (52.9)	123 (49.6)	96 (54.5)	84 (50.6)
Females	124 (47.1)	125 (50.4)	80 (45.5)	82 (49.4)
Age (months) range at inclusion	6.0–8.9	6.0–8.9	6.0–8.9	6.0–8.9
**Nutritional status**				
Stunting^2^	55 (20.9)	70 (28.0)	37 (21.0)	20 (12.0)
Underweight^2^	25 (9.5)	36 (14.5)	15 (8.5)	20 (12.0)
Wasting^2^	12 (4.6)	12 (4.8)	7 (4.0)	9 (5.4)
**Breastfeeding frequency**				
≥ 8 times/day	170 (64.6)	172 (69.4)	109 (61.9)	117 (75.0)*
< 8 times/day	93 (35.4)	76 (30.6)	67 (38.1)	39 (25.0)
**Started complementary feeding**				
Yes	254 (96.6)	236 (95.2)	170 (96.6)	156 (94.0)
No	9 (3.4)	9 (4.8)	6 (3.4)	10 (6.0)
**Illness at baseline**				
Yes	94 (35.7)	71 (28.6)	65 (54.2)	48 (46.6)
No	169 (64.3)	177 (71.4)	55 (45.8)	55 (53.4)
**Maternal data**				
Maternal age (yrs) (range)	18–44	18–44	18–44	18–44
Maternal education				
0–4 yrs (0–lower primary)	122 (46.4)	108 (43.5)	77 (45.2)	75 (43.4)
5–7 yrs (upper primary)	103 (39.2	109 (44.0)	76 (43.2)	72 (43.4)
≥ 8 yrs (lower secondary and tertiary)	38 (14.4)	31 (12.5)	23 (13.1)	19 (11.4)
Number of children (range)	1–9	1–9	1–9	1–9
**Household data**				
Mean (SD) household size	5.5 (2.1)	5.5 (2.1)	5.5 (2.1)	5.5 (2.2)
Household size (range)	3–10	3–10	3–10	3–10
Mean (SD) household poverty score	47.8 (11.7)	47.6 (11.4)	48.3 (11.9)	46.7 (11.2)
Mean (SD) sanitation score	7.2 (1.9)	7.3 (1.9)	7.4 (0.9)	7.2 (0.9)
Household head age (yrs) (range)	20–63	20–70	20–63	20–70
Household head education				
0–4 yrs (0–lower primary)	82 (31.2)	84 (33.9)	47 (26.7)	52 (31.3)
5–7 yrs (upper primary)	107 (40.7)	111 (44.8)	76 (43.2)	77 (46.4)
≥ 8 yrs (lower secondary and tertiary)	74 (28.1)	53 (21.4)	53 (30.0)	37 (22.3)

^1^Values are *n* (%) unless otherwise specified.

^2^Z-score values are < − 2 SD of the median WHO reference group.

^3^The variation in *n* is due to missing data.

*p < 0.05.

### Microbiota composition

**Figure 3:**
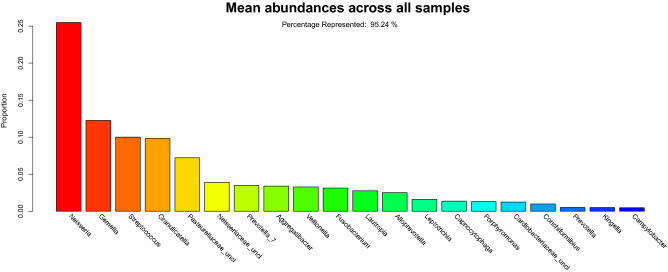
Overall rank abundance bar plot. The rank abundance plot illustrates the average abundance across all samples from the most abundant (*Neisseria*) to the least abundant (*Campylobacter*) across the top 20 taxa. The y-axis indicates the proportion of the taxa. The top 20 taxa represent 95.2% of the taxonomic classifications.

Relative abundance overall was dominated by organisms from the Firmicutes, Proteobacteria and Bacteroidetes phyla typically found in the oral cavity. The twenty most abundant taxa represented 95.2% of the taxa (Fig. 3).

### Effect of intervention on microbiota

**Figure 4:**
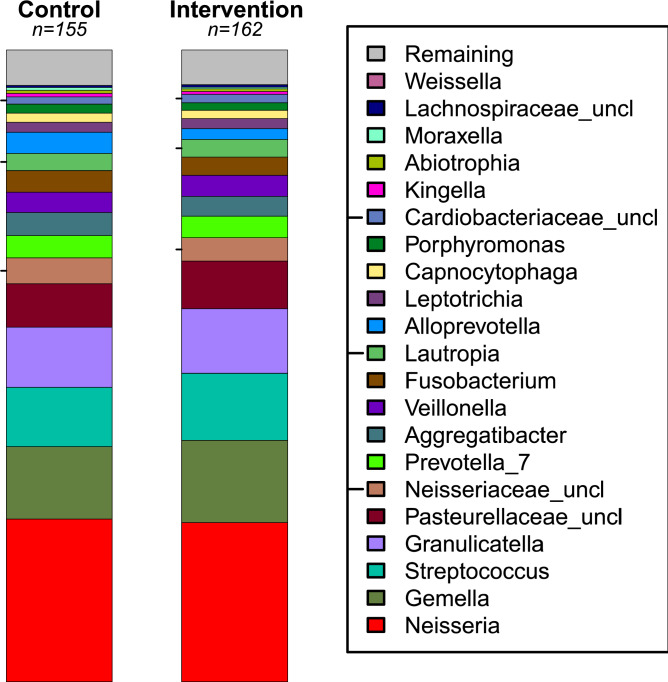
Stacked bar plot comparing control and intervention. The left and right stacked bar plots illustrate the average taxonomic composition of the control (155 subjects) and intervention (162 subjects) groups, respectively. The most and least abundant taxa are depicted at the bottom (*Neisseria*) and at the top (*Weissella*), respectively. The “Remaining” category is a placeholder for all the remaining taxa with abundances too low to represent. Tick marks placed on the left margin of the legend are also located by their corresponding position in the stacked bar plot. “Uncl” labeled taxa are reads not classifiable with confidence to the genus level.

**Figure 5:**
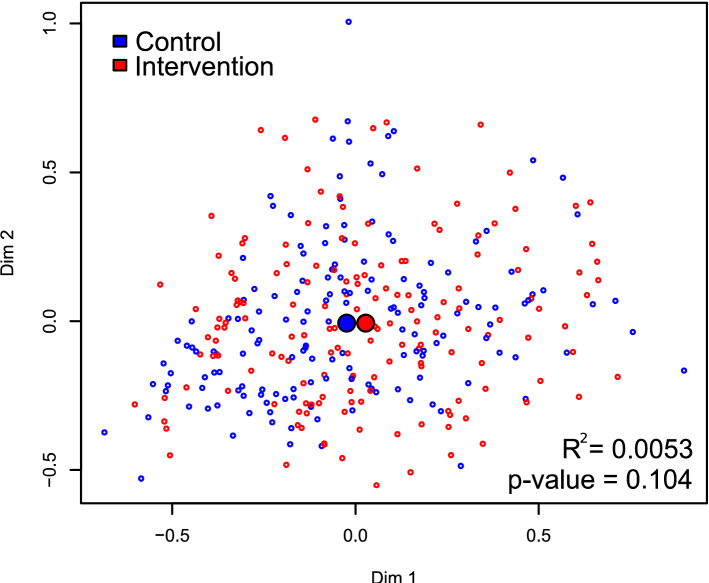
Multidimensional scaling (MDS) plot comparing control and intervention. The spatial separation between points represents the degree of the compositional Manhattan distance of the microbiota between samples. The x and y axes are unitless dimensions, although (0,0) represents the center of mass of all samples. Control and intervention samples are colored blue and red, respectively. The large blue and red circles represent the centroid of each group. PERMANOVA estimated a small difference (R^2^ = 0.0053) that was marginally statistically insignificant (*p*-value = 0.104).

There was no significant effect of the intervention on the Shannon diversity index (Fig. 4; *p* = 0.45). The PERMANOVA analysis identified a borderline effect between the control and intervention cohort (Fig. 5**;** R^2^ = 0.005, *p* = 0.10). There was also a statistically significant effect of age (R^2^ = 0.007, *p* = 0.034) and a borderline effect of fluoride (R^2^ = 0.006, *p* = 0.062). When examining the twenty most abundant taxa, the regression analysis associated intervention with an increased abundance of *Streptococcus* (coefficient = 0.20, *p* = 0.05) and a borderline increase of *Gemella* (coefficient = 0.19, *p* = 0.09) and a decreased abundance of *Alloprevotella* (coefficient = − 0.59, *p* < 0.01), and *Tannerella* (coefficient = − 0.50, *p* < 0.01) and a borderline decrease of *Fusobacterium* (coefficient = − 0.23, *p* = 0.07). Other associations with *p*-values < 0.05 were found with sex and age. See Supplementary Table S1 for additional information.

### Effect of microbiota on caries

There was no association between microbiota Shannon diversity and number of teeth with dentin caries (NTDC; *p* = 0.45) and number of teeth with severe tooth decay, (i.e. most severe diagnosis) (MSD; *p* = 0.39). The associations with dental health and the twenty most abundant taxa with *p*-values < 0.10 are reported. For NTDC, positive associations were identified with *Capnocytophaga* (coefficient = 0.24, *p* = 0.05) and negative associations with *Fusobacterium* (coefficient = − 0.17, *p* = 0.10). For MSD, positive associations were found with *Tannerella* (coefficient = 0.13, *p* = 0.08), and *Capnocytophaga* (coefficient = 0.26, *p* = 0.02), and negative associations were found with *Abiotrophia* (coefficient = − 0.11, *p* = 0.07), *Fusobacterium* (coefficient = − 0.17, *p* = 0.08) and *Granulicatella* (coefficient = − 0.16, *p* = 0.09). Of note, the associations with *Fusobacterium* and *Capnocytophaga* were shared between NTDC and MSD. When a multivariate ANOVA (MANOVA) was performed with MSD and NTDC as the multivariate response, an additional taxon, *Neisseria* (*p* = 0.07) was identified as borderline significant. *Granulicatella* (*p* = 0.07) and *Fusobacterium* (*p* = 0.08) were also identified to be borderline significant with the MANOVA, consistent with their identification in the univariate regressions. See Supplementary Table S2 for additional information.

### Effect of intervention and microbiota on caries

When controlling for microbial diversity, negative association trends were identified between intervention and the dental health variables: NTDC (coefficient = − 0.24, *p* = 0.078) and MSD (coefficient = − 0.21, *p* = 0.089). When the full model (with diversity) and reduced model (without diversity) were compared, the statistics indicated that the inclusion of this microbial diversity data did not improve the prediction of dental health (model ANOVA: NTDC; *p* = 0.74, MSD; *p* = 0.76).

In contrast, an examination of the model combining the influence of intervention and the twenty most abundant taxa revealed a statistically significant improvement of the model for both NTDC and MSD. The NTDC model’s adjusted R^2^ increased from 0.008 (reduced model: intervention) to 0.041 (full model: intervention + microbiota), a statistically significant improvement of the model (ANOVA comparison *p* = 0.091). Similarly, the adjusted R^2^ for MSD in the model increased from 0.012 to 0.056, with a model ANOVA comparison (*p* = 0.043). Examining the coefficients and *p*-values of the taxa between the full and reduced model, revealed that including the intervention had a small effect on the associations between taxa and oral health. When variables are added to an existing regression model, it is common that the associations of variables in the reduced model weaken (coefficients become closer to 0 and *p*-values increase) when the additional variables are correlated with the variables in the reduced model, or the additional variables are stronger predictors of the response (Supplementary Table S2).

## Discussion

Whereas the prevalence of caries has been markedly reduced among high-income countries from the 1970s, many African countries have faced an increase in caries during this time period^16,17^. Consequently, many vulnerable populations, in particular in sub-Saharan Africa suffer from poor oral health, that in turn may cause pain, impaired eating and thus quality of life. Since the microbiota may be different between e.g. American and African populations^18^, it is important to identify association between child microbiota and caries in African LMICs.

Consistent with previous studies, here we demonstrate that the saliva microbiota profile of children was associated with dental caries (a proxy for dental health)^19^. We used three statistical models to examine the effects of the (i) intervention on microbiota, (ii) microbiota on caries, and (iii) the combination of intervention and microbiota on caries. Collectively, the results identified several important findings regarding the effects of the educational intervention on child dental caries a low resource-setting.

First, the intervention had a significant effect on the microbiota composition, while alpha diversity was unchanged. Three taxa were identified as significant with increased abundances of *Streptococcus* and decreased abundances of *Alloprevotella* and *Tannerella* in the intervention relative to the control group and borderline associations with *Gemella* and *Fusobacterium*. Second, significant positive associations between the microbiota and dental health were identified (taxa abundance increased with increased NTDC and MSD) with *Capnocytophaga* and *Tannerella,* suggesting that these taxa may be deleterious to dental health. Concomitantly, significant negative associations between the microbiota and dental health were identified (taxa abundance decreased with increased NTDC and MSD) with *Granulicatella*, *Fusobacterium*, and *Abiotrophia,* suggesting these taxa may be benign or beneficial contributors to dental health. As with the first model, alpha diversity was not significantly associated with dental health.

The associations between microbiota and intervention (first model) and microbiota and caries (second model), collectively determined a complex and polymicrobial set of microorganisms commonly found in the oral microbiota. These taxa have been associated with both dental caries and periodontitis, suggesting potential underlying mechanistic relationships^20–22^.

*Streptococcus* and *Gemella*, (increased abundances in the intervention group) are both members of the phylum *Firmicutes* that share several general features: Both are frequently characterized as facultative anaerobes and can ferment sugars to organic acids that can demineralize enamel and interact with host defenses. Members of the genus *Gemella* can produce an IgA1 protease capable of degrading host secretory Immunoglobulin A (sIgA), a property that may enhance biofilm formation by conferring the capacity to circumvent the adherence-inhibitory activity of sIgA^23^. Since we examined oral microbiota, the increased abundances of *Streptococcus* and *Gemella* may in part reflect increased removal from tooth and gum surfaces in the intervention group.

The Gram negative *Bacteroidetes*, *Tannerella* and *Alloprevotella* and *Fusobacterium* (Fusobacteria) (decreased in intervention group) are functionally diverse, but often noted as proteolytic bacteria. As such, they can cause damage to tissue directly through the production of proteases such as collagenase and hyaluronidase and they can participate in the inflammatory response^21^. The best studied member of the genus *Tannerella* is *T. forsythia* and is considered a member of the “red complex” frequently associated with periodontitis^22^. *Alloprevotella* is an obligate anaerobe, non-spore-forming, rod-shaped and non-motile bacterium that has been isolated from both healthy oral and intestinal microbial communities. *Alloprevotella* can also produce organic acids such as acetic and succinic acids due to saccharolytic capabilities^24^.

From the second model, *Capnocytophaga* (Bacteroidetes) (increased abundance with caries) are noted for their growth requirements of high carbon dioxide level (at least 5%) and enriched media and have been associated with periodontal disease^25^. Like *Gemella*, *Abiotrophia* and *Granulicatella* (taxa abundance decreased with increased caries), are Gram-positive Firmicutes frequently characterized as facultative anaerobic bacteria that can ferment sugars to organic acids. However, members of these genera are also classified as nutritionally variant streptococci characterized by specific vitamin and amino-acid requirements for slower growth times^26^. Therefore, their decreased abundance may also be benign due to fastidious growth requirements that may make them less competitive with other caries producing taxa.

From the third model, which examined the effects of intervention and microbiota on dental health, a lack of significant overlap was determined between the taxa identified in the first model (those taxa modulated by intervention) and the second model (those taxa associated with dental health). This finding suggests that the outcomes from the educational intervention and the microbiota may largely have had independent effects on child dental health as measured by dentin caries (NTDC) and the most severe caries diagnosis (MSD) at 36 months of age.

One interpretation of our findings is that the effects of intervention on dental health acted with greater strength on host physiological processes compared to its influence on the saliva microbiota. Alternatively, the effects of the intervention may be more influential on microbiota function, which was not measured here. Although the taxa identified in the first two models (taxa modulated by intervention and taxa associated with dental health) differed, they are nonetheless ubiquitous members of the oral microbiota that at least broadly share multiple functional features, further emphasizing that functional changes in the microbiota may be of particular relevance. Moreover, saliva as a sample represents a mix of microorganisms detached from oral surfaces. Other ecological niches of the oral cavity, such as biofilms from soft epithelium or teeth, could more accurately reflect changes in microbiota composition due to the educational intervention. Notably, saliva was collected by sponges because of the children´s low age. Thus, it cannot be ruled out, due to cooperation challenges, that some samples may have contained more directly components of the oral mucosa and/or teeth including microorganisms from these biofilms. Moreover, possible cavities in posterior teeth were not registered. The current study lacks microbiota-data and information on dietary intake at the start of the RCT until the children became 36 months. A further limitation is the impossibility to detect initial caries on the photographs and thus associated primary colonizers. Notwithstanding this, important strengths of our study are the large sample size and availability of data form a robustly designed RCT in a challenging low-resource setting.

As we previously reported, despite the inclusion of a nutrition component in the education intervention, poor nutritional status was not significantly different between the intervention and control groups^8,11^. Furthermore, there was no evidence of any association between the occurrence of caries and child growth^9^. In contrast to our current saliva-results, a microbiota examination conducted in fecal specimen collected from these children when they were 20–24 and at 36 months, showed no statistically significant impact on gut microbiota composition at either time point^11^.

Our current study suggests a complex functional interplay between the saliva microbiota and host. Beneficial effects may occur through competitive or cooperative interactions between microbiota, and microbiota acting as physical and biochemical barriers to host epithelium, while conversion of dietary sugars and carbohydrates to organic acids within biofilms can create local decreases in pH that contribute to caries formation^27,28^. Given the importance of diet on the structure and function of oral microbiota and the apparent independence between intervention and microbiota effects on dental health, our results suggest that additional optimization of the nutrition component of the intervention could improve beneficial effects on the microbiota in the context of dental health.

In conclusion, we identified a significant effect on the saliva microbiota of children participating in a maternal education intervention focusing on nutrition (oral) hygiene and child stimulation. We also identified potentially beneficial, or benign to adverse associations between microbiota and caries, coupled to overall less caries in the intervention group. The educational intervention and the microbiota may have had independent effects on dental health or alternatively, additional examinations such as measures of biological function or other oral ecological niches, may be required to better elucidate educational intervention and microbiota interactions.

## Supplementary Information

Supplementary Information.

## Data Availability

Data described in the manuscript, code book, and analytic code will be made available upon request pending application and approval. 16S rRNA gene sequence data can be found online at https://www.ncbi.nlm.nih.gov with the BioProject ID: PRJNA834828 at the time of manuscript publication.
