# Supplementary material for: Child saliva microbiota and caries: a randomized controlled maternal education trial in rural Uganda
Source: Sci Rep. 2022 May 12;12:7857. doi: 10.1038/s41598-022-11979-y (PMC9098507; doi:10.1038/s41598-022-11979-y)
Supplement: Supplementary file 1 — Supplementary Information. [file 41598_2022_11979_MOESM1_ESM.pdf]

Supplementary Information

Child saliva microbiota and caries - a randomized controlled maternal education trial in rural Uganda

Grace K.M. Muhoozi[1,12], Kelvin Li[2,12], Prudence Atukunda[3], Anne B. Skaare[4], Tiril Willumsen[4], Morten Enersen[5,] Ane C. Westerberg[6,7], Alison Morris[2,8], Alexandre R. Vieira[9], Per O. Iversen[3,10,11,*] & Barbara A. Methé[2,8]

[1]Department of Family Life and Consumer Studies, Kyambogo University, Kampala, Uganda.
[2]Center for Medicine and the Microbiota, University of Pittsburgh, Pittsburgh, USA.
[3]Department of Nutrition, Institute of Basic Medical Sciences, University of Oslo, Oslo, Norway.
[4]Institute of Clinical Dentistry, Institute of Oral Biology, Faculty, of Dentistry, University of Oslo, Oslo Norway.
[5]Institute of Oral Biology, Faculty, of Dentistry, University of Oslo, Oslo Norway
[6]Institute of Health Sciences, Kristiania University College, Oslo, Norway.
[7]Division of Obstetrics and Gynecology, Oslo University Hospital, Oslo, Norway.
[8]Department of Medicine, Division of Pulmonary, Allergy and Critical Care Medicine, University of Pittsburgh, Pittsburgh, USA.
[9]Department of Oral & Craniofacial Sciences Biology, School of Dental Medicine, University of Pittsburgh, Pittsburgh, USA.
[10]Department of Haematology, Oslo University Hospital, Oslo, Norway.
[11]Division of Human Nutrition, Stellenbosch University, Tygerberg, South Africa.
[12]These two authors contributed equally to the manuscript.

Table of Contents

Supplementary Methods:

- *Randomization and sample size calculation ……………………………………………page 3*
- *The education intervention in the original cluster-randomized controlled trial………….4*
- *Booster sessions of the educational components after the intervention period……….….7*
- *Routine health care practices…………………………………………………………….....7*
- *Processing of oral samples for 16SrRNA sequencing……………………………….……8*
- *Bioinformatics…………………………………………………………………………….……8*
- *Analyses of microbiota profiles…………………………………………………………...9*
- *Microbiota and intervention models……………………………………………………….10*
- *Multiple statistical testing………………………………………………………………..11*
- *References…………………………………………………………………………………….12*

Supplementary Table S1……………………………………………………………..*14*

Supplementary Table S2……………………………………………………………..*15*

Supplementary Methods

Randomization and sample size calculation

The original cluster-randomized controlled trial (RCT) was performed between October 2012 and February 2014. Proportionate sampling was used to obtain 10 sub-counties (i.e. clusters; six of 19 in Kabale and four of 14 in Kisoro) to participate in the trial. Districts are made up of several sub-counties, and each sub-county is an administrative unit consisting of 18–25 villages. Town centers within the districts were excluded to minimize differences in socio-economic status, oral hygiene and feeding practices. Households were excluded if the child had (i) congenital malformation(s), (ii) a physical disorder that would influence growth or preclude anthropometric measurements or influence nutrient intake, (iii) been diagnosed with a mental or brain illness as reported by the mother or a health worker, (iv) if the household was likely to migrate within the study period, or (v) if the mother was unable to provide information or unwilling to participate in the study. The study personnel collecting the data and analyzing the study outcomes were blinded to group allocation.

We used a three-stage procedure to obtain households for the trial. First, by simple random sampling, sub-counties in both districts were allocated to the intervention or control group. Second, all the villages in each participating sub-county (intervention or control) were listed alphabetically and assigned numbers in ascending order. By use of computer-generated random numbers, villages whose position matched with the random numbers were identified eligible. Third, by complete enumeration, all consenting households with children aged 6–8 months within a participating village were recruited to the study.

In the original cluster-RCT, the sample size calculation was based on the primary outcome which was height-for-age z-score (HAZ) at child age of 20–24 months. We defined a difference of 0.3 SD in HAZ between the intervention and control group as clinically relevant, corresponding to about half a percentile in HAZ [1]. To detect a change of 0.3 SD in HAZ with a significance level of 5% and a power of 80%, 176 children were required per group. Fifty-one children per sub-county were included presuming 10 sub-counties as clusters and an intra-cluster correlation (ICC) of 0.01 [2]. To also account for dropouts etc., we ended up by including 511 mother–child pairs, and the assessment was by intention-to-treat.

For the current follow-up study, we decided to include as many children as possible from the original cluster-RCT, since attrition rates can be challenging in such low-resource settings with the study-sites in sometimes difficult topography. From the sample size calculation given above we arrived at a minimum number of 2 x 176 = 352 children to reach the primary outcome in the

original cluster-RCT. In the current follow-up study we were able to include 174 (intervention group) + 170 (control group) = 344 children.

The education intervention in the original cluster-randomized controlled trial

An education intervention emphasizing nutrition, hygiene (including oral hygiene) and stimulation was delivered to mothers in the intervention group. No intervention-related harm was detected. Cooking and oral hygiene demonstrations together with making of play toys to promote child stimulation were parts of the education intervention package. The intervention lasted six months in which each group of mothers received three main education sessions (with a nutrition education team) followed by monthly mother group meetings to remind them of key aspects of the intervention. Our strategy with the intervention was to promote behaviour change through providing information and prompt practice (demonstrations). The intervention is detailed below.

<u>Nutrition education</u>

The nutrition package was centred on PAHO/WHO guiding principles of complementary feeding of a breastfed infant (quality and quantity of complementary feeds) [3]. The main emphasis was on:

- The importance of breastfeeding and a demonstration of how to position and attach the infant to the breast.
- The need to allow emptying one breast before changing to the other breast so that the infant could benefit from both the fore and hind breast milk.
- Breastfeeding eight or more times in a day including at night.
- All mothers were asked to start complementary feeding if they had not done so, since all infants were between 6 and 8 eight months of age.
- In complementary feeding, they were advised to start with soft foods in small amounts at a time and gradually increase the portion and the thickness of the food.
- Providing food that is rich in variety of nutrients and the importance of combining a variety of foods in one dish.
- To give infants complementary foods 2-3 times a day and increase the frequency of feeding to 3-4 times a day as the child grew.
- Providing nutritious healthy snacks (such as fruit) to the infant in between the main meals.
- Interaction and responsiveness while feeding the infants by talking, smiling and

encouraging them to eat more without forcing them; to exercise patience and make feeding session a time for joy and bonding.

- To allow the infants to eat finger foods which they could hold with their hands.
- Continued breastfeeding until the child was 24 months of age.
- Breastfeeding more frequently, providing more fluids during illness (especially in diarrhoea and fever) of the infant, and giving foods that are more nutritious after recovery.

<u>Cookery demonstrations</u>

The cookery activities involved:

- Dishes which could combine up to 13 different foods in one obtained in their local environments.
- Inexpensive formulated recipes using locally available foods with emphasis on animal protein obtained from silverfish (*Rastrineobola argentea)* locally known as *Mukene.*
- Soy milk making, scraping meat (muscle), preparation of pumpkin seed powder and silverfish powder to incorporate in the infant's food, addition of oil/fat and sugar to porridges to increase the energy content.
- Preparation of enriched porridge recipe 1 and 2 which were enriched with the ingredients of; cooking oil, sugar, silver fish powder, milk, pumpkin seed powder and eggs; in combinations of two or more.
- Preparation of scrambled eggs preferred to the boiled eggs or omelette, which are rather hard for the infants to consume.

<u>Hygiene education</u>

Themes of emphasis included:

- The importance of living in a clean home environment for the good health of the family particularly the young children.
- The basic requirement to always wash hands and utensils with clean water and soap during food preparation and infant feeding.
- The prerequisite to clean food before preparation to make it free of soil and other contaminants.
- Mothers were encouraged to carry water and a piece of soap to the field/gardens to wash hands before feeding the infants.

- Mothers were warned on giving leftover foods to the infants, since safety of such food was not possible and safe for the infants to consume later.
- Licking spoons as they fed the babies (to test the temperature) was discouraged to avoid transmission of infections from the mother to the infant.

Child stimulation

The child play and stimulation emphasized:

- The importance of age graded child play activities and the role of mothers, other family members to engage in child stimulation.
- The significance of play to promote healthy development of the child.
- Explanation of the three development domains (cognitive, language and motor domains).

We explained to the mothers that the aim of play was to develop imagination creativity and social skills in the child [4]. The mothers were encouraged to use “name and identify” child’s body parts to facilitate the child’s understanding during his/her daily routine related to his body [5]. Practically, mothers engaged children in some of child play activities such as hiding favourite items for children to find; screwing and unscrewing bottles and imaginary play. Mothers also hand-made “easy to make” toys (from local materials) which were recommended as appropriate for children; shakers, empty transparent bottles with screws and food pellets inside, baby dolls made from cloth or banana fibres.

Language development was defined as verbal and non-verbal communication (expressive and receptive language) [6]. “We Talk” slogan was used to show mothers the importance of talking to the child so that they learn to talk back, and in the process, develop language skills [6]. Mothers were encouraged using communication development aides such as imitation, roleplaying games, songs and music, to facilitate the child’s ability to communicate emotions, thoughts, needs and interests [7,8]. The mothers were encouraged to set aside time to purposefully talk to the children, call them by their name and to respond to them in word and/by gesturing; mention household and personal items while pointing at them, naming domestic animals, imitating their words and actions.

For motor development, the “Learn whereas playing” slogan was emphasized. The concept of gross motor skills was explained as the use of coordination and control of the body to facilitate the development of security, speed, and accuracy [9] in daily performance of tasks in a child’s life (larger movements like walking and kicking). Fine motor skills were defined as the ability

to perform complex skills for more proficient tasks of daily living [7] (smaller movements like writing, tying shoelaces, and unbuttoning clothes). The following activities were emphasized:

- Giving child items to hold with their fingers, for example handing a pencil and paper for them to scribble.
- Matching lids with same size colour and shape games.
- Threading with beads
- Poking straws into holes.
- Stacking cups

The recommended toys included balls, bottle lids, cups, big beads, threads, ropes, shakers, pencils and paper. Furthermore, the mothers were encouraged to empower each other, by meeting regularly in their groups to practice and evaluate their childcare skills. We also advised them to be active with their sub-county activities for easy identification by government programs targeting women.

Booster sessions of the educational components after the intervention period

To prolong the effects of, and adherence to, the education intervention after the 6-months' intervention period had ended and until the children were aged 36 months, we administered booster sessions to groups of 6-12 women from the original trial cohort of 511 women. These sessions (each lasting about 6 hours) were provided by the education team every third month and started three months after end of the intervention period, hence a maximum of 8 booster sessions were given. The sessions were reminders of the education activities taught during the intervention period and re-emphasised the importance of (i) making nutritious meals; (ii) hand-washing and hygienic preparations, and (iii) child stimulation.

Routine health care practices

The intervention group received routine health care and the education intervention while the control group received only routine health care. The routine health care consisted of the recommended regular anthropometric measurements, immunizations, deworming, vitamin A supplementation, malaria-prophylaxis and iron-deficiency anemia prevention. Importantly, when the children were aged 20-24 months we found that mothers in the intervention group had gained significantly more knowledge and better practices related to child feeding, hygiene and stimulation [10] compared to the control mothers, indicating that the contents of our education intervention differed markedly from routine health care.

Processing of oral samples for 16SrRNA gene sequencing

Genomic DNA extractions were performed using the Qiagen DNeasy Powersoil Kit (Qiagen, Germantown, MD) and processed per manufacturer's protocol. Reagent blanks were included as negative controls and genomic DNA from a microbial community of known composition (ZymoBiomics Microbial Community Standards; Zymo Research, Irvine, CA) was included as a positive control.

The V4 region of the 16S rRNA gene was amplified in triplicate from each saliva sample with inline barcoded primers whose design was based on the method of Caporaso [11]. Approximately 5-10 ng of genomic DNA from each sample was used as template in each amplification in 25 µl volume reactions. Amplifications were performed using Q5 HS High-Fidelity polymerase (New England BioLabs, Ipswich, MA). Primer sequences for the 16S rRNA gene V4 region used were: 515f 5'-GTGCCAGCMGCCGCGGTAA-3' and 806r 5'-GGACTACHVGGGTWTCTAAT-3'. Cycle conditions were 98ºC for 30s, then 30 cycles of 98º C for 10 s, 57 ºC for 30 s, and 72 ºC for 30 s, with a final extension step of 72 ºC for 2 min. Amplicons were purified with AMPure XP beads (Beckman Coulter, Indianapolis, IN) at a 0.8:1 ratio (beads:DNA) to remove primer-dimers. Eluted DNA was quantitated on a Qubit fluorimeter (Life Technologies, Grand Island, NY).

Sample pooling was performed on ice by combining 40 ng of each purified band. For negative controls and poorly performing samples, 20 µl of each sample was used. The sample pool was purified with the MinElute PCR purification kit (Qiagen, Germantown, MD). The final sample pool underwent two more purifications – AMPure XP beads to 0.8:1 to remove primer dimers and a final cleanup in Purelink PCR Purification Kit (Life Technologies Cat #K310001; Grand Island, NY). The purified pool was quantitated in triplicate on the Qubit fluorimeter prior to sequencing. The sequencing pool was prepared as per Illumina's recommendations, with an added incubation at 95ºC for 2 minutes immediately following the initial dilution to 20 pM. The pool was then diluted to a final concentration of 7 pM + 15% PhiX control. Sequencing was done on an Illumina MiSeq 500-cycle V2 Nano kit (Illumina, Inc, San Diego, CA).

Bioinformatics

Sequences from the Illumina MiSeq were deconvolved and then processed through the Center for Medicine and the Microbiota (CMM) in-house sequence quality control pipeline, which includes dust low complexity filtering, quality value trimming, and trimming of primers used for 16S rRNA gene amplification, and minimum read length filtering. Forward and reverse reads were merged into contigs then processed through the CMM's Mothur-based [12] 16S

clustering and sequence annotation pipeline. Sequence taxonomic classification was performed with the Ribosomal Database Project Naive Bayesian Classifier with the Silva reference database [13, 14].

Sequences from each technical replicate per sample were processed through the Mothur-based pipeline and taxonomic profiles were generated for each replicate. Replicate compositions were examined for consistency through PERMANOVA. Profiles from each technical replicate classification were merged into a single profile and this single profile was used for subsequent analyses. To validate consistency between sequence runs we randomly selected amplicon libraries for repeat sequencing on subsequent runs. These run-to-run technical replicates were examined for technical consistency using PERMANOVA but were not used in the subsequent statistical analyses.

We then developed three linear regression models to examine the relationship between educational intervention, microbiota, and dental health. Additional details on the analyses of microbiota profiles including this modelling are given below and in Figure 1 in the main text file.

Analyses of microbiota profiles

Targeted Variables: The variables included in the linear regression model were the educational intervention, and the covariates of fluoride concentration in drinking water, sex, and age, and oral (dental) health status of the children quantified by "the number of teeth with dentin caries" (NTDC) and "most severe diagnosis" (MSD) at 36 months of age identified with photographs. From the 16S rRNA gene sequence clustering and annotation pipeline, samples were generated and subsequently analyzed.

There were three categories of microbiota analyses, distribution-based, distance-based, and abundance-based, that were performed on the taxonomic profiles:

Distribution-based analyses: The distribution-based analyses consider the entire composition of taxa within a sample with a single metric, such as the Shannon diversity index (higher values denote more diversity), and samples are compared to each other with this computed value. Alpha (i.e. diversity within a single sample) diversity indices are useful quantifications of compositional data represented visually as stacked barplots and rank abundance plots.

Distance-based analyses: The distance-based analysis computes the pairwise difference between two samples profiles using the Manhattan distance, then subsequent analyses focus on the association of variables with clustering or linear models. Permutational multivariate

analysis of variance (PERMANOVA) was performed with the "adonis" function from the "vegan" library in R to associate targeted variables with inter-sample distances and test for their statistical significances [15, 16].

Abundance-based analyses: The abundance-based analyses provide a means to consider each taxon as an independent and normally distributed entity after they have been transformed with the additive log ratio (alr) transformation [17]. This reduces the spurious correlations between taxa that occurs due to the compositional nature of 16S rRNA gene sequence profiles, and the transformation is necessary for each taxonomic category to be considered as independent predictors in a multiple regression or as a multivariate response [18]. The twenty most abundant taxa, by average abundance across all samples, were selected for inclusion into the regression models. The remaining taxa not in the top were accumulated into the remainder of the log ratio.

Microbiota and intervention models

Three models were considered to ascertain the relationship between educational intervention, microbiota, and oral (dental) health. These models are as follows: (i) effect of intervention on microbiota, (ii) effect of microbiota on oral health, and (iii) effect of intervention and microbiota on caries.

(i) Effect of intervention on microbiota: This model identifies how the microbiota profiles from subjects are different between the control and intervention cohorts, while controlling for the covariates (age, sex and fluoride). The microbiota metrics are considered responses to the intervention as a predictor (treatment).

(ii) Effect of microbiota on oral health: This model identifies the association between the microbiota and oral health, without controlling for intervention. The microbiota profiles are utilized, along with the covariates (age, sex and fluoride) as predictors of caries at 36 months. The intervention is excluded from the model because if the intervention had an effect on taxa associated with oral health, then the affected taxa and intervention would be confounded.

(iii) Effect of intervention and microbiota on caries: This model includes both the intervention variable and the microbiota profiles (and covariates) in the model so that the contributions of both factors can be cumulatively considered. This model also provides model statistics to determine whether the inclusion of the microbiota profiles improves the predictability of oral health in contrast to intervention alone. To estimate

the contribution of the microbiota to oral health, a full (microbiota + intervention) and reduced (intervention only) model are fit and compared to each other.

Multiple statistical testing

Correcting for multiple testing is important when asserting whether a proportion of tests or a specific set of tests of interest are simultaneously significant in the context of the total number of tests performed to identify the associations. We have chosen not to report corrected p-values for multiple testing since this prohibits the reader from using their own judgement when determining which set of tests are of interest. For example, predictors, such as covariates, whose statistical significance may not be of central importance to a hypothesis, nonetheless have values that need to be estimated in the model to control for them. There may be disagreement as to which clinical variables collected to characterize a disease are of interest, and since the observations may only be different manifestations of the same disorder, penalizing all p-values for each symptom's association is inappropriate. In addition, microbiota data is compositional in nature and there is a relationship between assay reliability and abundance, so not all p-values for each taxonomic association should be considered equivalent or of interest. In general, when analyzing groups of related variables, we examine MANOVA p-values to assert whether a collection of variables responds to a set of predictors, but the underlying univariate associations are important to examine independently, so these are also reported.

References

1. Ong, K. K., et al., *Association between postnatal catch-up growth and obesity in childhood: Prospective cohort study*. BMJ, 2000. 320: 967–971.
2. Campbell, M. J., et al., *Developments in cluster randomized trials and Statistics in Medicine.* 2007. Statistics in Medicine, 26: 2–19.
3. PAHO/WHO, *Guiding Priciples for Complementary Feeding of the Breastfed Child.* Division of Health Promotion and Protection, 2003(Washington D.C.).
4. Bruner, J.S. and H. Haste, *Making Sense (Routledge Revivals): The Child's Construction of the World*. 2010: Routledge.
5. Chang, S.M., et al., *Integrating a parenting intervention with routine primary health care: a cluster randomized trial.* Pediatrics, 2015. 136: 272-280.
6. Vos, R.C., et al., *Developmental trajectories of receptive and expressive communication in children and young adults with cerebral palsy.* Dev Med Child Neurol, 2014. 56: 951-959.
7. Hartinger, S.M., et al., *Impact of a child stimulation intervention on early child development in rural Peru: a cluster randomised trial using a reciprocal control design.* J Epidemiol Comm Health, 2017. 71: 217-224.
8. Wolf, J., et al., *Impact of a child stimulation intervention on early child development in rural Peru: a cluster randomised trial using a reciprocal control design.* 2017. J Epidemiol Community Health, 71: 217-224.
9. Donnelly, J.E., et al., *Physical activity, fitness, cognitive function, and academic achievement in children: a systematic review.* Med Sci Sports Exerc, 2016. 48: 1197.
10. Muhoozi, G.K., et al., *Nutrition, hygiene, and stimulation education to improve growth, cognitive, language, and motor development among infants in Uganda: A cluster-randomized trial.* Matern Child Nutr, 2018. 14: e12527.
11. Caporaso, J. G., et al. *Ultra-high-throughput microbial community analysis on the Illumina HiSeq and MiSeq platforms*. ISME J, 2012. 6(8): 1621-1624.
12. Schloss, P.D., et al. *Introducing mothur: Open-source, platform-independent, community-supported software for describing and comparing microbial communities.* Applied and Environmental Microbiology, 2009. 75:7537–7541.
13. Wang, Q., Naïve Bayesian *Classifier for Rapid Assignment of rRNA Sequences into the New Bacterial Taxonomy*. Appl Environ Microbiol, 2007. 73(16):5261-5267; doi: 10.1128/AEM.00062-07. PMID: 17586664.
14. Cole, J. R., *Ribosomal Database Project: data and tools for high throughput rRNA*

analysis. Nucl. Acids Res., 2014. 42(Database issue):D633-D642; doi: 10.1093/nar/gkt1244. PMID: 24288368.

15. Oksanen, J. et al., *vegan: Community Ecology Package. R package* version 2.5-3, 2018. Available at: https://CRAN.R-project.org/package=vegan
16. R Core Team., *R: A language and environment for statistical computing*. R Foundation for Statistical Computing, Vienna, Austria, 2019. Available at: https://www.R-project.org/
17. Aitchison, J. *Principles of compositional data analysis.* Lecture Notes-Monograph Series. 1994. PP. 73-81.
18. Gloor, GB. et al.; *Microbiota datasets are compositional: and this is not optional.* Front Microbiol, 2017.8:2224.

Supplementary Table S1. Coefficients and P-values for Effect of Intervention on Microbiota

Coefficient and p-values cells were highligted in pink if p-values < 0.10.

Model 1: Effect of Intervention on Microbiota

Coefficients

	Fluoride_X	is Intervention	is Female	age_yrs
Neisseria	0,1595	0,0114	0,1423	0,0957
Gemella	0,0706	0,1890	0,1988	0,4174
Streptococcus	-0,0502	0,1989	0,0687	-1,0576
Granulicatella	0,1008	0,1132	0,2026	0,0264
Pasteurellaceae_uncl	0,1735	0,1351	0,1931	0,4974
Neisseriaceae_uncl	-0,0048	-0,0780	0,0804	0,1851
Prevotella_7	0,2175	-0,1051	0,0392	-0,1111
Aggregatibacter	-0,0024	-0,0076	0,0272	-0,3214
Veillonella	0,0226	0,0258	-0,0107	-1,1559
Fusobacterium	0,0928	-0,2307	0,0267	0,5557
Lautropia	-0,1476	0,2203	0,3251	-1,8522
Alloprevotella	-0,1252	-0,5894	-0,1989	-0,3054
Leptotrichia	-0,1869	0,0447	-0,0558	-1,2037
Capnocytophaga	0,0104	-0,1457	-0,0629	0,1633
Porphyromonas	0,0135	-0,2076	0,3880	-0,5077
Cardiobacteriaceae_uncl	-0,0991	0,1459	0,0613	-0,6619
Conchiformibius	-0,0216	-0,1112	0,1887	0,5271
Prevotella	0,0323	-0,1445	-0,1308	0,0153
Kingella	-0,0241	-0,0931	0,0972	-0,8009
Campylobacter	-0,0533	-0,0627	0,0341	-0,5541
Abiotrophia	0,1561	-0,0044	0,2269	0,8253
Bergeyella	-0,1853	0,1560	-0,0326	0,0839
Tannerella	-0,0145	-0,4978	-0,0385	-0,4018
Lachnoanaerobaculum	0,0218	-0,0780	0,0672	-0,2093
Moraxella	0,1386	0,0083	0,2127	0,5628

P-values

	Fluoride_X	is Intervention	is Female	age_yrs
Neisseria	0,0831	0,8961	0,1028	0,7958
Gemella	0,5530	0,0941	0,0784	0,3836
Streptococcus	0,6404	0,0515	0,5004	0,0150
Granulicatella	0,4625	0,3837	0,1196	0,9619
Pasteurellaceae_uncl	0,1377	0,2221	0,0815	0,2901
Neisseriaceae_uncl	0,9617	0,4098	0,3958	0,6454
Prevotella_7	0,1690	0,4822	0,7932	0,8613
Aggregatibacter	0,9856	0,9520	0,8302	0,5504
Veillonella	0,8642	0,8369	0,9323	0,0307
Fusobacterium	0,4883	0,0696	0,8332	0,3027
Lautropia	0,2986	0,1019	0,0161	0,0013
Alloprevotella	0,5476	0,0030	0,3137	0,7155
Leptotrichia	0,1211	0,6951	0,6251	0,0134
Capnocytophaga	0,9308	0,2007	0,5805	0,7355
Porphyromonas	0,9360	0,1939	0,0156	0,4542
Cardiobacteriaceae_uncl	0,6364	0,4626	0,7576	0,4331
Conchiformibius	0,8704	0,3759	0,1336	0,3233
Prevotella	0,8294	0,3093	0,3576	0,9798
Kingella	0,8769	0,5278	0,5100	0,2018
Campylobacter	0,6416	0,5632	0,7535	0,2297
Abiotrophia	0,3025	0,9753	0,1143	0,1760
Bergeyella	0,1629	0,2149	0,7956	0,8751
Tannerella	0,9254	0,0008	0,7934	0,5197
Lachnoanaerobaculum	0,8565	0,4949	0,5565	0,6664
Moraxella	0,4737	0,9638	0,2464	0,4700

Supplementary Table S2. Coefficients and P-values for Effect of Microbiota on Caries

Coefficient and p-values cells were highligted in pink if p-values < 0.10

Model 2: Effect of Microbiota on Oral Health

Coefficients	NTDC	MSD	P-values	NTDC	MSD	MANOVA P-values	MANOVA
Neisseria	-0,106	-0,139	Neisseria	0,471	0,304	Neisseria	0,0727
Gemella	-0,048	-0,010	Gemella	0,658	0,924	Gemella	0,1377
Streptococcus	-0,122	-0,108	Streptococcus	0,293	0,312	Streptococcus	0,4447
Granulicatella	-0,077	-0,161	Granulicatella	0,459	0,092	Granulicatella	0,0721
Pasteurellaceae	0,108	0,054	Pasteurellaceae	0,327	0,589	Pasteurellaceae_un	0,7788
Neisseriaceae_u	-0,076	0,006	Neisseriaceae_u	0,620	0,964	Neisseriaceae_uncl	0,3716
Prevotella_7	0,044	0,023	Prevotella_7	0,591	0,762	Prevotella_7	0,8797
Aggregatibacter	0,125	0,131	Aggregatibacter	0,161	0,109	Aggregatibacter	0,1984
Veillonella	0,118	0,120	Veillonella	0,212	0,168	Veillonella	0,6879
Fusobacterium	-0,174	-0,166	Fusobacterium	0,100	0,085	Fusobacterium	0,0804
Lautropia	-0,065	-0,038	Lautropia	0,447	0,626	Lautropia	0,9609
Alloprevotella	0,018	-0,018	Alloprevotella	0,722	0,696	Alloprevotella	0,6122
Leptotrichia	-0,046	-0,037	Leptotrichia	0,648	0,687	Leptotrichia	0,5832
Capnocytophaga	0,236	0,258	Capnocytophaga	0,048	0,019	Capnocytophaga	0,2054
Porphyromonas	-0,074	0,000	Porphyromonas	0,250	0,993	Porphyromonas	0,1165
Cardiobacteriac	0,052	-0,009	Cardiobacteriac	0,351	0,867	Cardiobacteriaceae_	0,1122
Conchiformibius	-0,072	-0,077	Conchiformibius	0,443	0,374	Conchiformibius	0,7998
Prevotella	-0,073	-0,071	Prevotella	0,395	0,365	Prevotella	0,6842
Kingella	0,096	0,073	Kingella	0,122	0,197	Kingella	0,2734
Campylobacter	-0,107	-0,138	Campylobacter	0,291	0,138	Campylobacter	0,3553
Abiotrophia	-0,084	-0,114	Abiotrophia	0,223	0,071	Abiotrophia	0,187
Bergeyella	-0,107	-0,071	Bergeyella	0,175	0,323	Bergeyella	0,4152
Tannerella	0,122	0,128	Tannerella	0,127	0,081	Tannerella	0,252
Lachnoanaeroba	-0,124	-0,043	Lachnoanaeroba	0,241	0,653	Lachnoanaerobacul	0,3068
Moraxella	0,060	0,049	Moraxella	0,219	0,269	Moraxella	0,4678

Model 3: Effect of Intervention + Microbiota on Oral Health *(Full Model)*

	NTDC	MSD		NTDC	MSD		MANOVA
Neisseria	-0,116	-0,146	Neisseria	0,432	0,280	Neisseria	0,0744
Gemella	-0,042	-0,005	Gemella	0,703	0,964	Gemella	0,1968
Streptococcus	-0,126	-0,111	Streptococcus	0,277	0,299	Streptococcus	0,5018
Granulicatella	-0,073	-0,158	Granulicatella	0,486	0,100	Granulicatella	0,0656
Pasteurellaceae	0,121	0,064	Pasteurellaceae	0,272	0,524	Pasteurellaceae_un	0,7769
Neisseriaceae_u	-0,076	0,006	Neisseriaceae_u	0,618	0,965	Neisseriaceae_uncl	0,3965
Prevotella_7	0,038	0,018	Prevotella_7	0,643	0,809	Prevotella_7	0,844
Aggregatibacter	0,128	0,134	Aggregatibacter	0,150	0,102	Aggregatibacter	0,199
Veillonella	0,117	0,119	Veillonella	0,214	0,169	Veillonella	0,7134
Fusobacterium	-0,173	-0,166	Fusobacterium	0,100	0,085	Fusobacterium	0,0575
Lautropia	-0,054	-0,030	Lautropia	0,524	0,699	Lautropia	0,9641
Alloprevotella	0,011	-0,023	Alloprevotella	0,832	0,614	Alloprevotella	0,5032
Leptotrichia	-0,040	-0,033	Leptotrichia	0,688	0,721	Leptotrichia	0,6432
Capnocytophaga	0,222	0,247	Capnocytophaga	0,065	0,025	Capnocytophaga	0,2488
Porphyromonas	-0,079	-0,005	Porphyromonas	0,218	0,939	Porphyromonas	0,1072
Cardiobacteriac	0,061	-0,002	Cardiobacteriac	0,283	0,963	Cardiobacteriaceae_	0,101
Conchiformibius	-0,083	-0,085	Conchiformibius	0,379	0,327	Conchiformibius	0,7105
Prevotella	-0,066	-0,066	Prevotella	0,444	0,404	Prevotella	0,7433
Kingella	0,094	0,072	Kingella	0,131	0,208	Kingella	0,3026
Campylobacter	-0,104	-0,136	Campylobacter	0,307	0,146	Campylobacter	0,3851
Abiotrophia	-0,081	-0,112	Abiotrophia	0,241	0,077	Abiotrophia	0,1987
Bergeyella	-0,099	-0,065	Bergeyella	0,211	0,368	Bergeyella	0,493
Tannerella	0,103	0,114	Tannerella	0,205	0,127	Tannerella	0,3245
Lachnoanaeroba	-0,115	-0,037	Lachnoanaeroba	0,275	0,701	Lachnoanaerobacul	0,3373
Moraxella	0,057	0,047	Moraxella	0,238	0,287	Moraxella	0,4956
